# Sodium oxybate – new views on an old candidate This presentation will outline the outcome of a clinical development program, including a Phase 3 study, on sodium oxybate in the treatment of alcohol dependence

**DOI:** 10.1192/j.eurpsy.2024.38

**Published:** 2024-08-27

**Authors:** J. Guiraud

**Affiliations:** ^1^Department of Psychiatry, Amsterdam UMC, University of Amsterdam, Amsterdam, Netherlands; ^2^Vergio, Paris, France

## Abstract

**Abstract:**

Sodium oxybate (SMO) has shown efficacy in the treatment of alcohol withdrawal syndrome (AWS) and in the maintenance of abstinence in alcohol dependent (AD) patients in a series of pilot randomized controlled trials. SMO is marketed in these indications in Italy and Austria since 1991 and 1999, respectively. To expand access to SMO for the treatment of AD in other EU countries and since regulatory standards have evolved, a clinical development and research project in accordance with regulatory guidelines has been initiated in the maintenance of abstinence to further support the already available data. Phase 2 and 3 studies in AD patients were conducted. Results of this development program showed efficacy of SMO in the maintenance of abstinence in AD patients. Since heterogeneity of SMO treatment effect between studies was identified, various analyses explored the potential moderators of SMO efficacy. SMO efficacy was larger in high-severity AD population and with longer treatment duration. SMO was well tolerated both in regular clinical use and in clinical trials.

**Disclosure of Interest:**

J. Guiraud Shareolder of: Vergio, Employee of: Vergio

